# Metallic glass coating for improving diamond dicing performance

**DOI:** 10.1038/s41598-020-69399-9

**Published:** 2020-07-24

**Authors:** Jinn P. Chu, Bo-Zhang Lai, Pakman Yiu, Yu-Lin Shen, Chia-Wei Chang

**Affiliations:** 1 Department of Materials Science and Engineering, National Taiwan University of Science and Technology, Taipei, 10607 Taiwan; 2 Applied Research Center for Thin-Film Metallic Glass, National Taiwan University of Science and Technology, Taipei, 10607 Taiwan; 3 Department of Mechanical Engineering, University of New Mexico, Albuquerque, NM 87131 USA

**Keywords:** Mechanical engineering, Mechanical properties

## Abstract

This is the first report on the coating of diamond dicing blades with metallic glass (MG) coating to reduce chipping when used to cut Si, SiC, sapphire, and patterned sapphire substrates (PSS). The low coefficient-of-friction (CoF) of Zr-based MG-coated dicing blades was shown to reduce the number and size of chips, regardless of the target substrate. Overall, SiC, sapphire and PSS were most affected by chipping, due to the fact that higher cutting forces were needed for the higher hardness of SiC, sapphire and PSS. Compared to the bare blade, the MG coating provided the following reductions in chipping area: Si (~ 23%), SiC (~ 36%), sapphire (~ 45%), and PSS (~ 33%). The proposed coating proved particularly effective in reducing chips of larger size (> 41 µm in chipping width), as indicated by an ~ 80% reduction when cutting sapphire. Small variations in kerf angle and depth demonstrate the durability of the coated blades, which would no doubt enhance consistency in dicing performance and extend the blade lifespan. Finite-element modeling revealed significant reductions in tensile stress and elastic–plastic deformation during dicing, thanks to a lower CoF.

## Introduction

Diamond abrasive blades are widely used to cut silicon, SiC, and sapphire dies in the manufacture of semiconductors and optoelectronic devices^1–3^. The brittleness of these hard workpieces inevitably leads to chipping and/or other types of damage, which might also give rise to failure in dies and chips, eventually causes yield loss^4–6^. Space between dies, called streets is usually spared to accommodate chipping on both sides of the dicing kerf. Yet this approach reduces the number of die per wafer and thus is a waste of very expensive substrate material. Researchers have sought to improve cutting quality by adjusting the concentration and size of diamond grit^7,8^ as well as the bonding material^7,9^. Note also that it is essential to minimize the number and size of chips due to the fact that even undetectable chipping provides sites for potential failure under the application of a strong electrical field. Controlling the factors that lead to chipping also has benefits for the environment, by reducing the amount of water required for cooling and decreasing the amount of waste debris.

This is the first report on coating diamond dicing blades with thin film metallic glass (TFMG) to reduce chipping when used to cut Si, SiC, sapphire, and patterned sapphire substrates (PSS). We focused on these workpieces due to their wide-scale use in semiconductors and light-emitting diodes. This is the first application of a coating on diamond dicing blades to enhance dicing performance. The thin film metallic glass (TFMG) coating in this study has a low coefficient of friction (CoF) and notable glass transition and crystallization characteristics^10,11^. Multicomponent amorphous metallic films are an attractive alternative to conventional alloys, thanks to their smooth surface, high hardness, and high ductility as well as low-temperature processing (e.g., sputter deposition). The low CoF (~ 0.05) and non-stick properties of TFMGs make them ideal coatings for knives and needles applied to biological materials^11–14^. In previous studies, we reported on the durability of these devices, as evidenced by their low cutting and insertion forces after being used multiple times. Compared with a bare blade, a blade coated with Teflon (e.g., microtome blades for thin slicing biological samples) can reduce cutting forces by ~ 80%. TFMG-coated blades can reduce cutting forces by ~ 51% without any of the peeling to which Teflon-coated blades are susceptible^13^.

In this paper, we turned our attention from soft materials to workpieces of extreme hardness and brittleness. In a previous paper^11^, we briefly touched on the benefits of using TFMG coatings to enhance blade dicing performance; however, this paper provides a thorough examination of the process and confirms the reproducibility of our results. We also conducted numerical modeling using the finite element method to gain insight into the issue of cracking during the dicing of hard substrate materials. The chipping induced by coated blades was assessed experimentally in terms of chip size and area fraction in the kerfs. We also measured the kerf depth and angle to evaluate the wear resistance of bare and coated blades.

## Experimental methods

The deposition of Zr-based TFMG coatings was performed under vacuum at base and working pressures of < 9.3 × 10^–4^ Pa and 5.0 × 10^–4^ Pa, respectively. We employed a high-power impulse magnetron sputtering system (HiPIMS, Highpulse Bipolar 4,002 G2, TRUMPF Huttinger, Germany) using 2.5 kW of power and a 6-inch alloy target. The working distance was 8.68 cm and the deposition rate was 6.07 nm/min. The nominal coating thickness was 200 nm. Using an electron probe micro-analyzer, we estimated the composition of the coating (in atomic percentage) as follows: Zr: 61.7 ± 0.2%, Cu: 24.6 ± 0.1%, Al: 7.7 ± 0.1%, and Ni: 6.0 ± 0.1%. Before and after the dicing experiments, the coating on the blade was examined using a scanning electron microscope (SEM) as part of a dual-beam focused-ion-beam system (FIB, FEI Quanta 3D FEG). FIB sectioning of the coated blade enabled observation of the coatings in cross section. The coating and coated blade were characterized for their crystallographic structures by an X-ray diffractometer (XRD, Bruker D8 DISCOVER) with monochromatic Cu Kα radiation.

**Figure 1:**
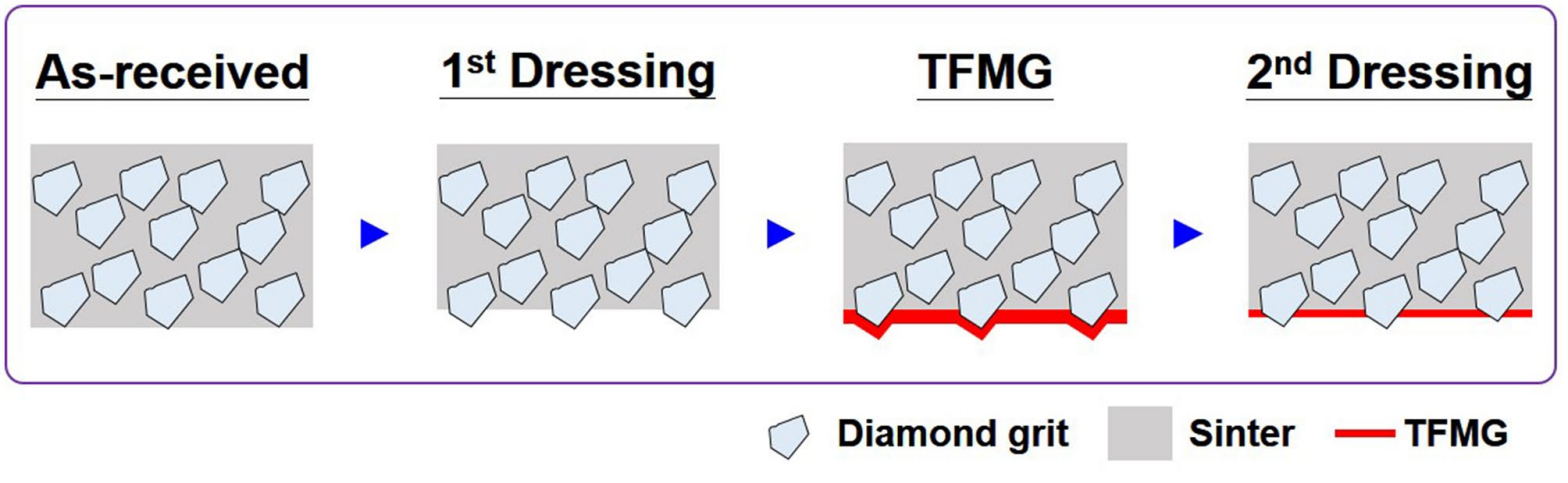
Schematic of preparation of diamond dicing blade. The first dressing is applied to as-received blade to expose diamond grits, followed by deposition of TFMG coating and the second dressing. The second dressing is applied at a small extent to remove the TFMG coating on diamond grits.

Details of the Si, SiC, sapphire and PSS workpieces are listed in Table 1. Unless otherwise mentioned, all dicing experiments in this study were conducted using a commercial Fe-Co-Sn sintered diamond dicing blade (Taiwan Asahi Diamond Industrial Co., Ltd.). The blade had a diamond grit size of 22/36 µm, outer diameter of 58 mm, blade thickness of 0.89 mm, and chamfering angle of 60°. For the sake of comparison, we also coated a blade with similar dimensions from another manufacturer (DISCO Corporation, Japan). Prior to TFMG deposition, the as-received blades were subjected to initial dressing to expose the diamond grit across the cutting area (as shown in Fig. 1) using a whetstone (WA600L, Asahi Diamond Industrial Australia Pty. Ltd.). The dressing was cut to a depth of 400 µm for 10 kerfs. After this first dressing, we applied the TFMG coating and then performed a light second dressing to remove all traces of TFMG from the diamond grit surface prior to dicing. Note that the bare blade did not undergo a second dressing. At least three samples from each type of workpiece underwent dicing using bare and coated blades. The spindle speed was maintained at 35,000 rpm during dressing and 25,000 rpm during dicing. All dressing/dicing processes were performed using an automated dicing saw system at a feed rate of 5 mm/s in down-cut mode using deionized water for cooling.

**Table 1:** Details of workpieces and dicing operation parameters.

Workpiece	Workpiece specification	Dicing operation			
		Direction	Depth (µm)	Width (µm)	Number of kerf
Silicon	525 µm thick, 4 in., (110) plane, P-type	< 110 >	400^b^	661.9	20
SiC	350 µm thick, 4 in., (0001) plane, 4H, N-type	$<11\stackrel{-}{2}0>$	200^c^	370	20
Sapphire/PSS^a^	430 µm thick, 2 in., (0001) plane	$<11\stackrel{-}{2}0>$	100	315	20

^a^For PSS, the pattern has height of 2.6 µm and diameter of 2.35 µm.

^b^400 µm-depth kerf consists of two consecutive 200 µm-depth dicing.

^c^200 µm-depth kerf consists of two consecutive 100 µm-depth dicing by DISCO blade [MBT-5885 SD800N50M51]. Si wafer was used for the first dressing and no second dressing was applied in this case.

**Figure 2:**
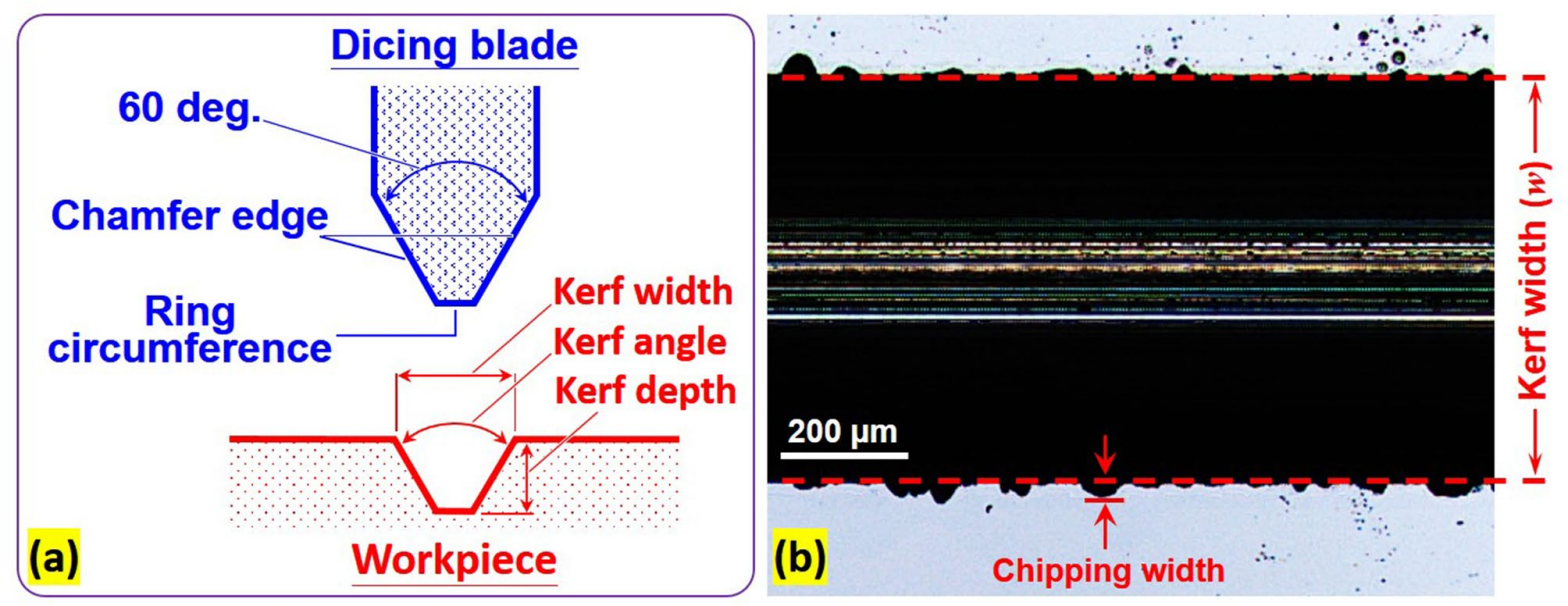
(**a**) Cross-sectional schematic of dicing blade and workpiece used in this study and (**b**) laser-confocal micrograph for measurements of kerf width and chipping width on the workpiece after dicing.

During dicing, the distance between the areas from which material was removed is referred to as the kerf width (*w*), as shown in Fig. 2a,b. The kerf angle and kerf depth are defined in Fig. 2a. Using a laser confocal microscope (OLS5000, Olympus), we measured the angle and depth of 20 kerfs, so the charts display that the angle and depth of 20 kerfs are on the corresponding abscissa of the cutting distance. The photograph of the kerf created when dicing the Si workpiece (Fig. 2b) shows the chipping width as observed through the laser confocal microscope. Image J and Image Pro software were used to calculate the area of the kerfs and the area affected by chipping. All measurements were derived as follows:

1 $$Area\left ( \%  \right) = \frac{{A_{R}  - w \times l}}{{w \times l}} \times 100,$$

where *Area (%)* refers to the fraction of the chipping area per kerf area, *A*_*R*_ is the dark area (including kerf and chipped area), *w* is the kerf width, and *l* is the distance along the midline of the kerf.

**Figure 3:**
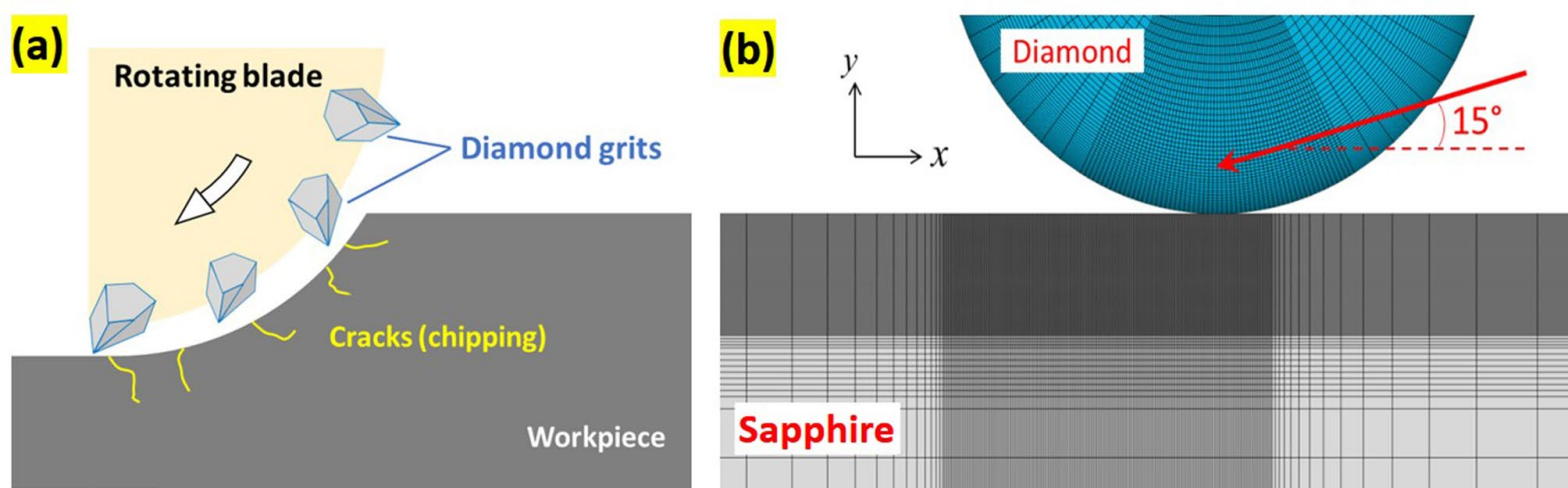
(**a**) Schematic showing the microscopic feature of the wafer cutting process. (**b**) Finite element model near the contact region between the particle and sapphire workpiece.

Numerical modeling was conducted using the finite element method. Figure 3a presents a schematic illustration showing a dicing blade with individual diamond particles traversing the surface of the workpiece. In this case, material removal is essentially a process of plowing by the grinding particles along with the sinter. We adopted a simplified simulation scheme for this analysis, wherein a single round domain with a diameter of 25 µm, featuring the property of diamond, was pressed onto a flat sapphire surface at an angle of 15°. The width of the sapphire was 1 mm and the thickness was 500 µm. Figure 3b shows the finite element mesh in the area surrounding the contact region. Four-node quadrilateral plane-strain elements were used to represent the particle and sapphire substrate, and extremely fine elements were allocated in the region of the sapphire adjacent to the scratch site. Movement of the round particle followed a prescribed displacement at a specified angle. During deformation, the bottom of the sapphire was not permitted to move in the *y*-direction. It was allowed to slide tangentially in the *x* direction; however, one corner point remained fixed.

The influence of the TFMG coating on dicing performance was simulated in terms of frictional characteristics^12^, wherein the reduction in friction qualitatively represented the effects of the coating. Based on Coulomb’s law of kinetic friction, we performed analysis using three CoF values between the particle and workpiece: 0.05, 0.2 and 0.5. Our numerical model did not account for material failure; however, the stress and deformation fields resulting from differences in CoF enabled a comparison of substrates based on their propensity to cracking.

We employed the finite element program ABAQUS (Dassault Systems Simulia Corp., Johnston, RI, USA) for our calculations. We first conducted baseline analysis assuming isotropic linear elastic behavior. The Young’s modulus *E* and Poisson’s ratio *ν* were as follows: *E*_diamond_ = 1,141 GPa, *ν*_diamond_ = 0.07, *E*_sapphire_ = 400 GPa and *ν*_sapphire_ = 0.29. This made it possible to use the maximum principal stress generated in the sapphire to assess the tendency toward cracking. We also explored one alternative approach in which we accounted for the plastic deformation of the substrate. This was motivated by experimental observations of twinning and/or slip leading to the initiation of fractures in sapphire^15–19^. In the proposed model, we adopted the compressive strength of sapphire (2,000 MPa) as the plastic yield strength, wherein a higher equivalent plastic strain under the effects of scratching indicated a higher propensity to failure.

## Results and discussion

**Figure 4:**
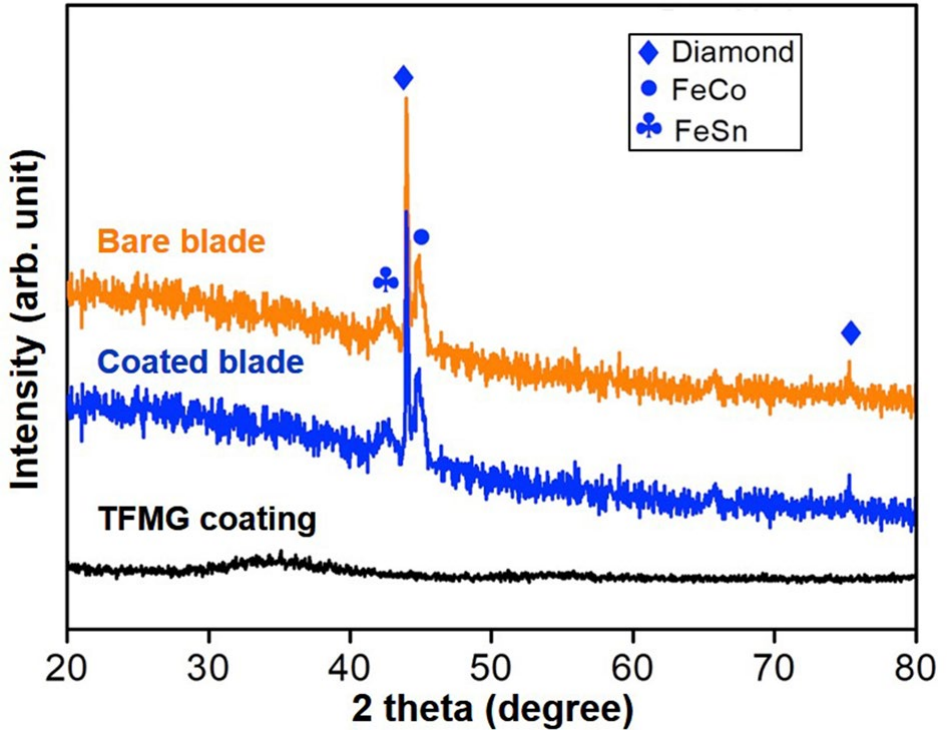
XRD patterns of TFMG coating, coated and bare blades. Bragg peaks from crystalline phases based on diamond, FeCo and FeSn are identified in both coated and bare blades.

**Figure 5:**
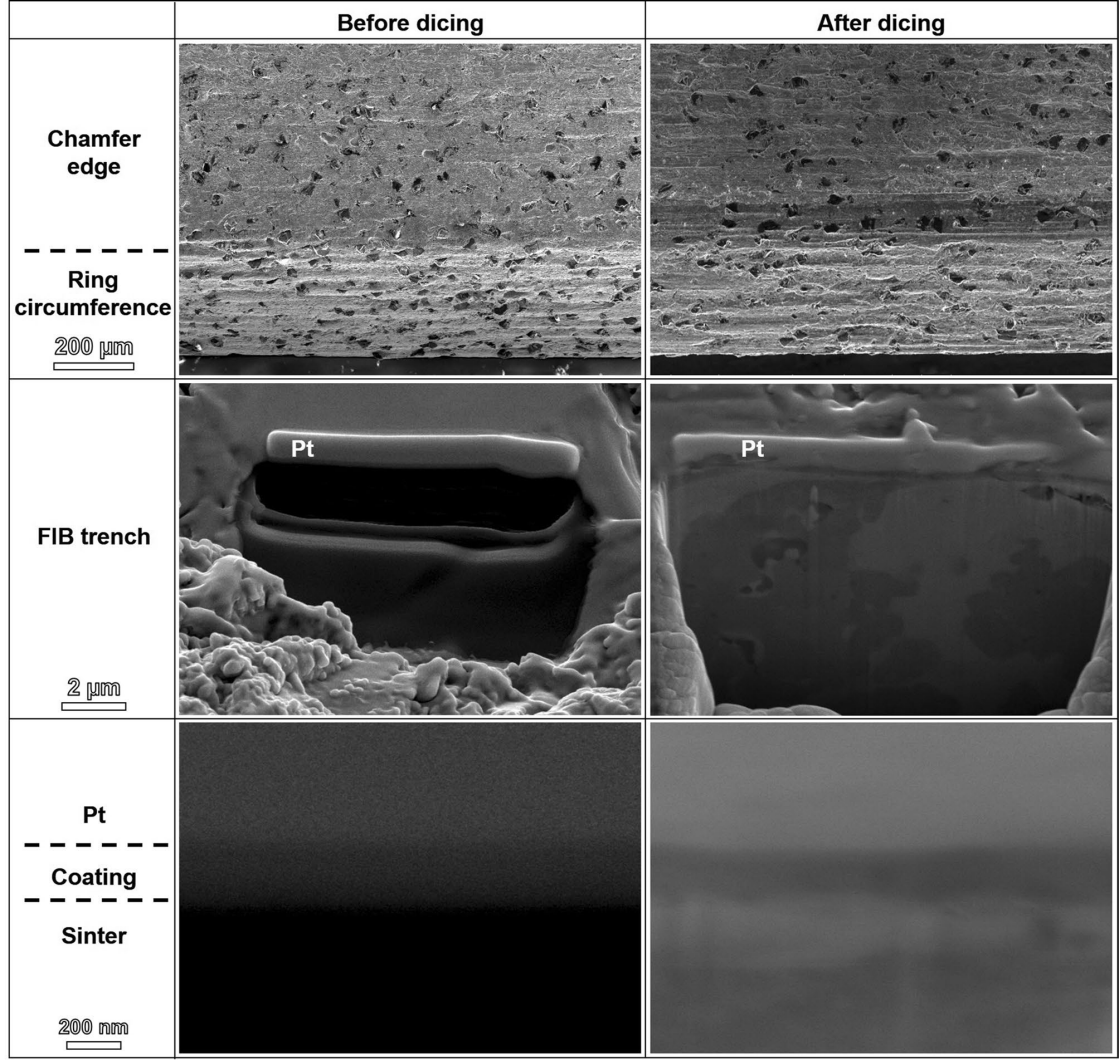
SEM micrographs of coated blades before and after dicing. Cross-sectional trenches were sectioned by FIB in the chamfer edge areas. Pt as the protective layer was deposited during FIB cutting.

Crystallographic analyses based on XRD confirmed the amorphous nature of the coating, with results similar to those reported in Ref.^12,13^. XRD patterns obtained from the TFMG coating monolayer, bare and coated blades are shown in Fig. 4. The hump between 28 and 42° of 2θ in the pattern of TFMG confirms its amorphous nature, whereas the patterns from the bare and coated blades suggest that the 200 nm-thick coating had no observable effect on the structure of the blade. Focused ion beam (FIB) characterization was used to evaluate the adhesion between the sintered blade and the coating, the results of which are presented in Fig. 5. Based on SEM cross-sectional images after FIB sectioning, the process of cutting 20 kerfs was shown to reduce the thickness of the coating from approximately 200 nm to ~ 155 nm. These results demonstrate that the adhesion between the coating and the sintered metal was very good, based on the fact that the coating remained firmly attached, even after the coating was worn down by roughly 25% under the harsh conditions of cutting a hard workpiece a distance of more than 2 m.

**Figure 6:**
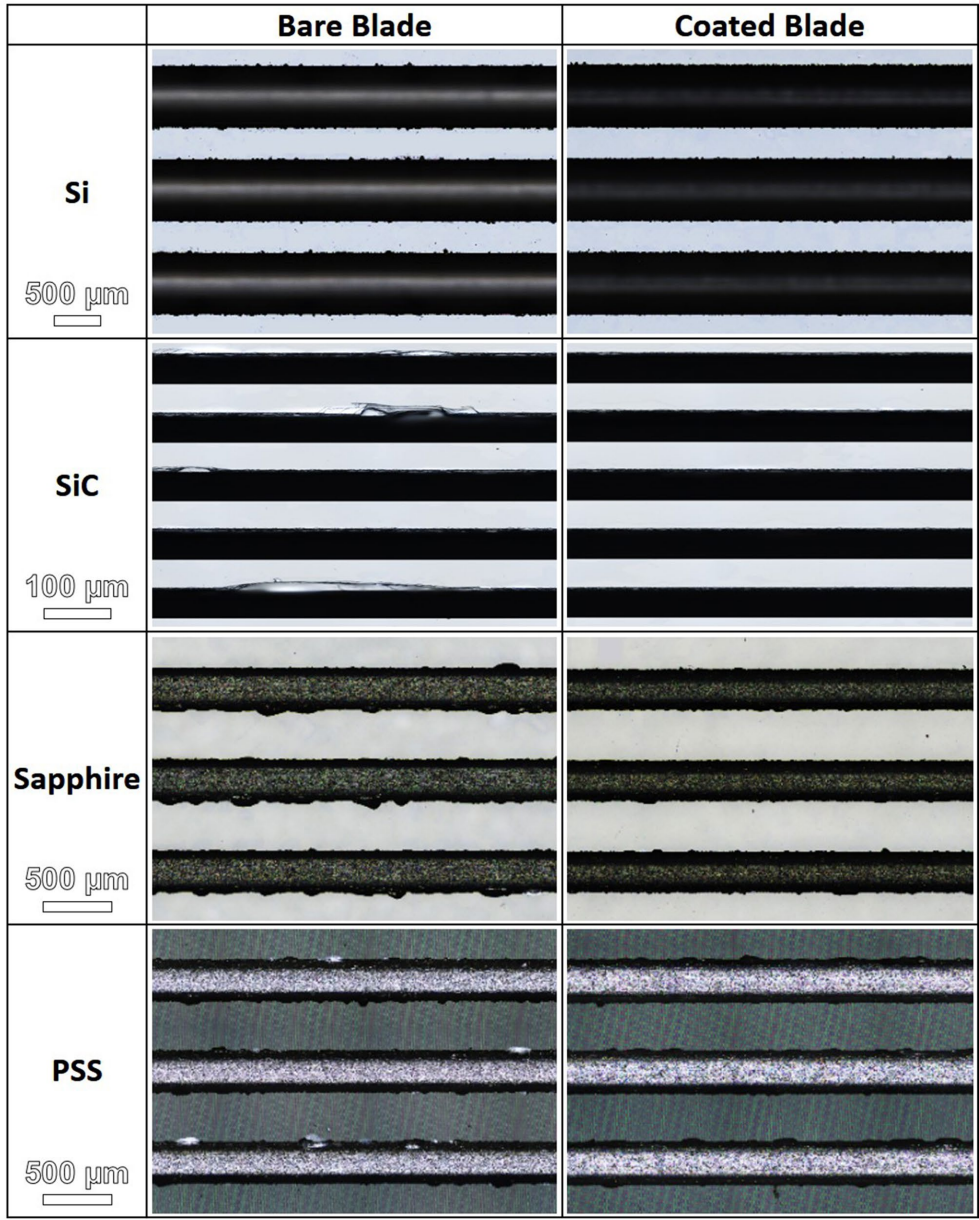
Laser confocal micrographs of selected kerfs from Si, SiC, sapphire and PSS workpieces after dicing with bare and coated blades. Note that micrographs for SiC are shown at a higher magnification.

**Figure 7:**
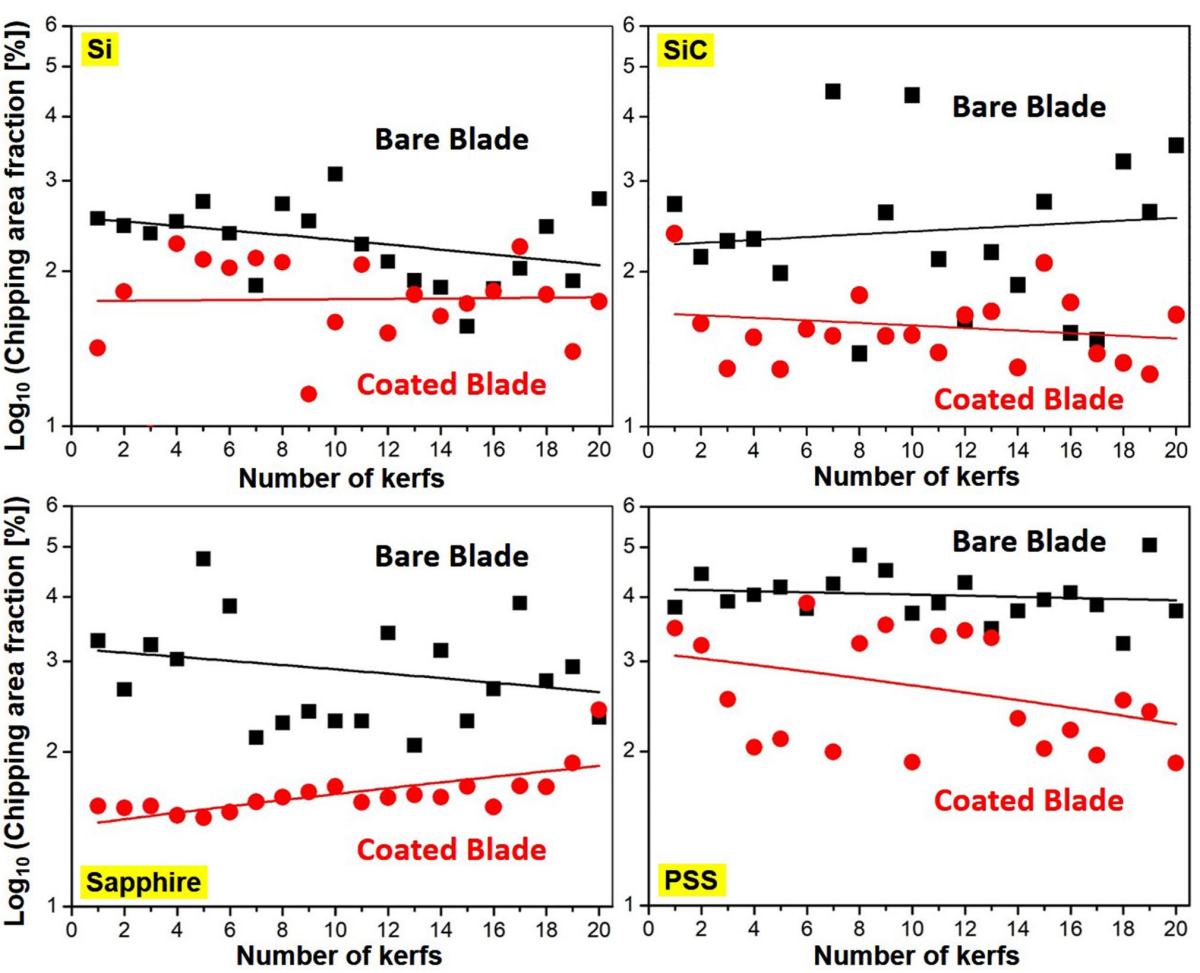
Chipping area fractions (in log scale) as a function of number of kerfs on Si, SiC, sapphire, and PSS workpieces after dicing with bare and coated blades.

The four workpieces were carefully observed using a laser confocal microscope after using bare or coated blades for dicing. Figure 6 presents typical laser confocal micrographs selected from the large-scale areas shown in Figs. S1–S4 in Supplement Information. As shown in Fig. 6, bare blades caused more chipping than did coated blades, regardless of the workpiece material. The area affected by chipping on the Si workpieces was smaller than on the SiC and sapphire workpieces, presumably due to the fact that Si lacks the hardness of SiC, sapphire and PSS, thereby allowing lower cutting forces. Note that the effects of the coating were more pronounced when applied to the harder materials (SiC, sapphire and PSS). We also plotted the fraction of the chipped area (on a log scale) as a function of the number of kerfs, as shown in Fig. 7. In these plots, the areas of chipping created by the coated blade were generally far smaller than those created by the bare blade. Compared to the bare blade, the TFMG coating provided the following reductions in chipping area: Si (~ 23%), sapphire (~ 45%), and PSS (~ 33%). On blades manufactured by DISCO, the coating performed very well when applied to Si and SiC workpieces. The coating reduced the chipping area fraction of the Si workpiece from 3.3% (bare blade) to 1.9% (coated blade), which represents an improvement of ~ 42%. The coating reduced the chipping area fraction of the SiC workpiece from 2.5% (bare) to 1.6% (coated blade), which represents an improvement of ~ 36%.

**Figure 8:**
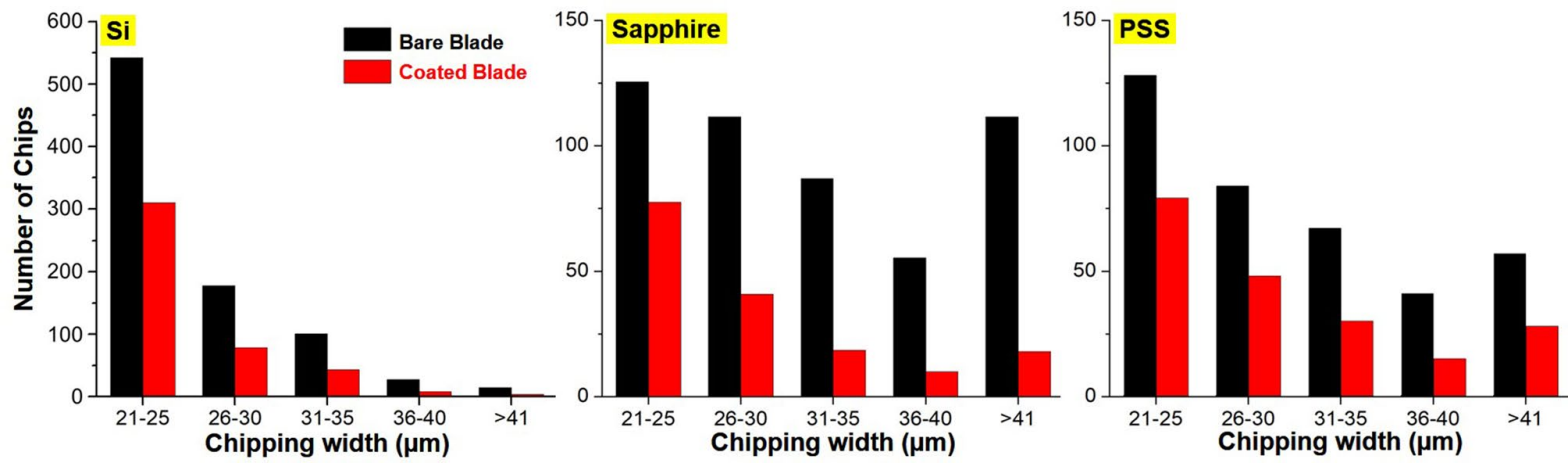
Number of chips as a function of chipping width in the range of 21 µm to over 41 µm for Si, sapphire and PSS after dicing with bare and coated blades.

Figure 8 plots the number of chips as a function of chipping width after using bare and coated blades for cutting. The width of the chips ranged from 21 to > 41 µm; however, we disregarded chips of less than 21 µm for brevity. The coated blade reduced ~ 43% of the chips 21–25 µm in width and ~ 70% of chips exceeding 41 µm in width for the Si. Note that when dicing the Si workpieces, a substantial proportion of the chips were of smaller size (21–25 µm). The size distributions of chips created from sapphire and PSS workpieces differed from those on the Si. Overall, there were slightly more mid-size chips (36–40 µm) on the sapphire than on the Si and far more large chips (> 41 µm). The coated blade consistently outperformed the bare blade, as evidenced by a notable reduction in the number of small- and mid-size chips and an enormous reduction (~ 80%) in the number of large chips. The large chip size (Fig. 8) and chipping area fraction (Fig. 7) of the sapphire and PSS substrates can be attributed to the large forces required to overcome their hardness.

**Figure 9:**
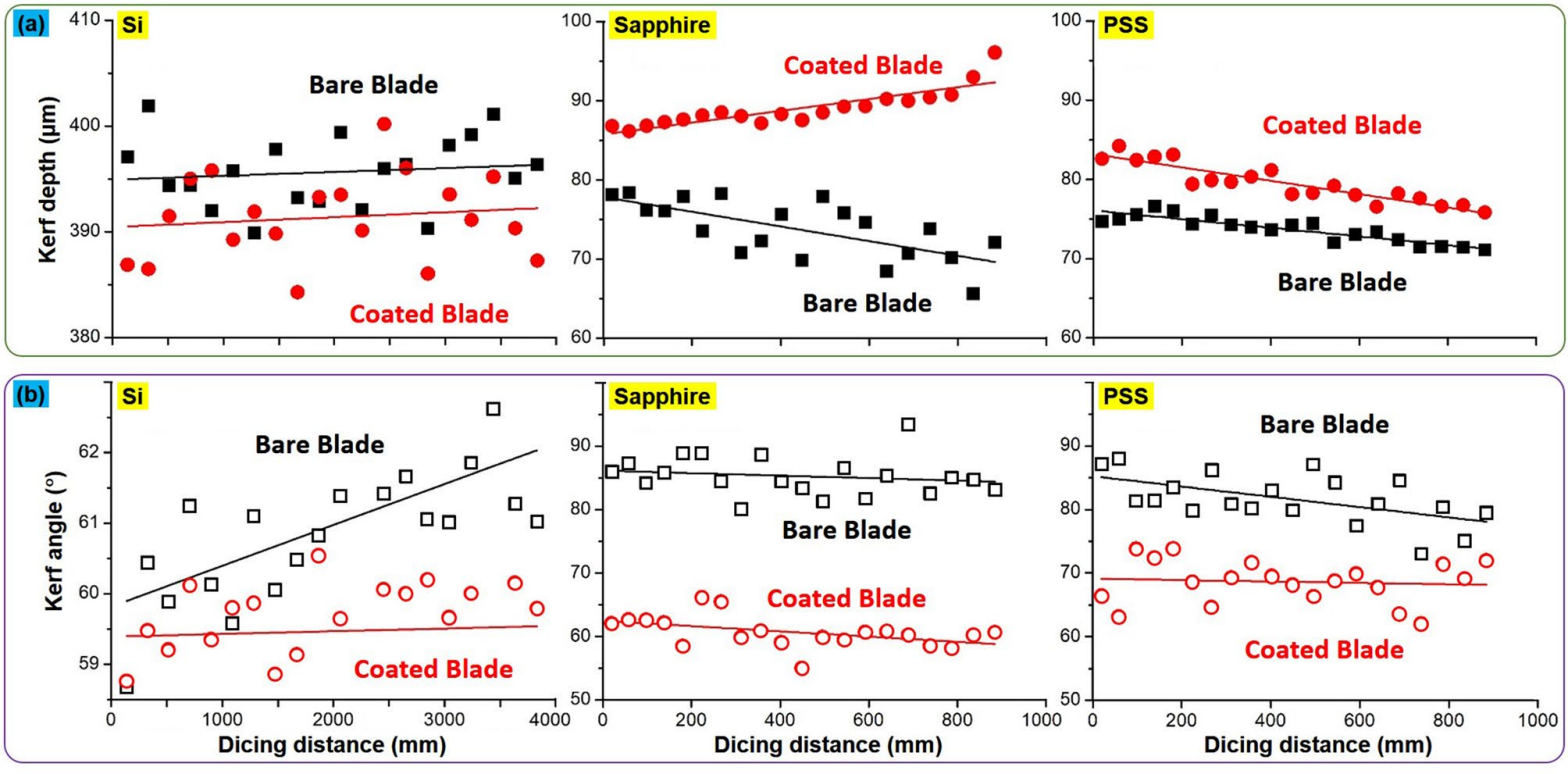
Changes in kerf depth (**a**) and kerf angle (**b**) as a function of dicing distance by bare and coated blades on Si, sapphire and PSS workpieces.

The performance of a dicing blade is expected to decay as a function of cutting distance. This process can be quantified by measuring variations in the kerf depth and kerf angle on workpieces. Figure 9a,b respectively plot changes in kerf depth and angle in the three workpieces as a function of cutting distance. When applied to the Si workpiece, the depth of the kerf created by the coated blade was shallower than that of the bare blade. Based on linear fitting, we estimated the difference at less than 5 µm. When applied to the sapphire, we noted a considerable difference between the coated and uncoated blades in terms of kerf depth. The depth of the kerf created by the uncoated blade was ~ 78 µm at the start of the cut, and ~ 65 µm at the end. By contrast, the depth of the kerf created by the coated blade increased slightly as the blade was advanced. When applied to the PSS workpiece, the depth of the kerf created by the coated blade was deeper than that of the bare blade throughout the entire cutting process.

The coating had more pronounced effects on kerf angle. Note that maintaining a consistent kerf angle is far more important than maintaining a consistent kerf depth. High kerf angles and changes in kerf angle are an indication of blade deterioration. An increase in kerf angle can lead to an increase in kerf width and perhaps a situation where the kerf depth is insufficient to penetrate the workpiece. When applied to Si workpieces, the kerf angle created by the bare blade increased with cutting distance, indicating that the blade was progressively wearing out. By contrast, the coated blade did not present significant changes in kerf angle. When applied to sapphire workpieces, the kerf angle created by the bare blade was roughly 20° larger than those created by the coated blade, throughout the entire cutting process. As shown in Fig. 9a, this increase in kerf angle led to a corresponding decrease in kerf depth. When applied to PSS workpieces, the kerf angle created by the bare blade was roughly 10° larger than that created by the coated blade. Overall, there were no significant variations in the kerf angle created by the coated blade, regardless of the material in the workpiece. This is a clear indication that the coated blades are well-suited to cutting hard Si and sapphire substrate materials.

When applied to two types of dicing blade from two different vendors, our results clearly demonstrate the benefits of the TFMG coating in reducing the number and size of chips from four workpieces. These benefits can be attributed primarily to the high hardness, good ductility, and low CoF of the coating. The hardness and ductility of the TFMG coating help to impede blade deterioration in terms of kerf depth and angle during dicing operations (Fig. 9). The fact is that the hardness of the coating exceeds that of the sintered metal in the blade. Thus, the coating is able to shield the metal from wear and tear and thereby increase its service life (Fig. 5). The ductile coating is also able to deform with the sintered metal during dicing, thereby remaining adhered to the substrate and avoiding delamination^20^. Note however that the benefits of the coating in the term of chipping area fraction and kerf depth were not as pronounced when applied to Si workpieces. This is presumably because most of the chips (> 60%) were less than 25 µm, as shown in Fig. 8. The benefits of the TFMG coating became obvious when applied to harder materials, such as SiC, sapphire, and PSS, where 50% of the chips exceeded 25 µm.

**Figure 10:**
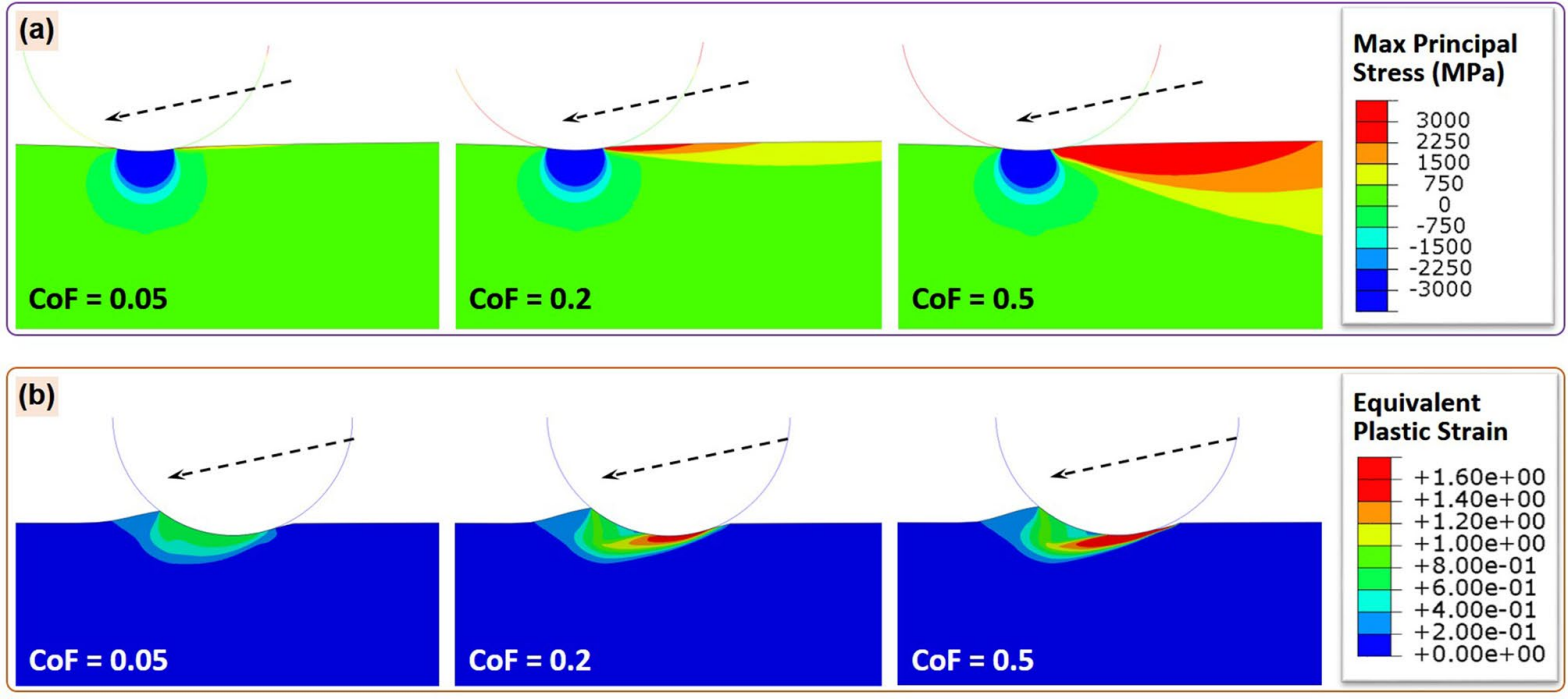
Contour plots of (**a**) maximum principal stress in the pure elastic models and (**b**) equivalent plastic strain in the elastic–plastic models, when the horizontal scratch distance reaches 11.2 µm, with the CoF values of 0.05, 0.2 and 0.5.

Our numerical modeling provided notable insights into the mechanisms underlying the propensity to chipping (cracking) during diamond cutting. The baseline elastic analysis is considered first. Figure 10a presents contour plots showing the maximum principal stress after the diamond particle traversed a horizontal distance of 11.2 µm (corresponding to a vertical depth of 3.0 µm) using CoF values of 0.05, 0.2 and 0.5. The dashed arrows indicate the direction of particle movement. These results indicate that directly beneath the contact region, the highest principal stress was compressive in nature, due to a severe triaxial compressive state locally. However, tensile stress components appeared close to the surface of the substrate opposite to the direction of the moving particle (i.e., ahead of the cutting blade as it moved), as shown in Fig. 10a. A further examination of the modeling results (not shown here) revealed that the highest principal stress was associated primarily with the high tensile *σ*_*xx*_ stress component associated with the dragging action. Tensile stress decreased dramatically with a decrease in the CoF, which appears to reduce the likelihood of cracking. This observation is consistent with our experiment results indicating that the low friction of the TFMG-coated blade reduced the incidence of chipping.

Our second numerical simulation involved elastic–plastic deformation. Figure 10b presents contour plots of equivalent plastic strain after the diamond particle traversed a horizontal distance of 11.2 µm under CoF values of 0.05, 0.2 and 0.5. The plastic deformation of the substrate material led to considerable pile-up effects in front of the moving particle. Extensive plastic deformation occurred beneath the contact region, resulting in the formation of a concentrated band parallel to the direction of the scratch. The accumulated plastic strain decreased with a decrease in the CoF, thereby reducing the likelihood of damage initiation. Our results in Fig. 10a,b provide a mechanistic rationale for the improvements in cutting performance provided by the coated blade.

## Summary

This study provided empirical evidence that the reduced CoF provided by a TFMG coating can greatly improve the cutting performance of diamond dicing blades when applied to Si, SiC, sapphire, and PSS. The Si substrate was least affected by chipping, due to the fact that it lacks the hardness of sapphire and PSS, such that it yields to lower cutting forces. Compared to the bare blade, the TFMG coating provided the following reductions in chipping area: Si (~ 23%), SiC (~ 36%), sapphire (~ 45%), and PSS (~ 33%). The proposed coating proved particularly effective in reducing chips of larger size (> 41 µm in kerf width) with an ~ 80% reduction when cutting sapphire. Small variations in kerf angle and depth demonstrate the durability of the coated blades, which would no doubt enhance consistency in cutting performance and extend the blade lifespan. The results of finite-element modeling revealed noticeable reductions in tensile stress and elastic–plastic deformation when using the coated blade, both of which can presumably be attributed to the low CoF (0.05).

## Supplementary information

Supplementary Information 1.
