# Supplementary material for: Metallic glass coating for improving diamond dicing performance
Source: Sci Rep. 2020 Jul 24;10:12432. doi: 10.1038/s41598-020-69399-9 (PMC7381673; doi:10.1038/s41598-020-69399-9)
Supplement: Supplementary file 1 — Supplementary Information 1. [file 41598_2020_69399_MOESM1_ESM.doc]

**Supplementary Information**

**Metallic Glass Coating for Improving Diamond Dicing Performance**

Jinn P. Chu, Bo-Zhang Lai, Pakman Yiu, Yu-Lin Shen, Chia-Wei Chang

**
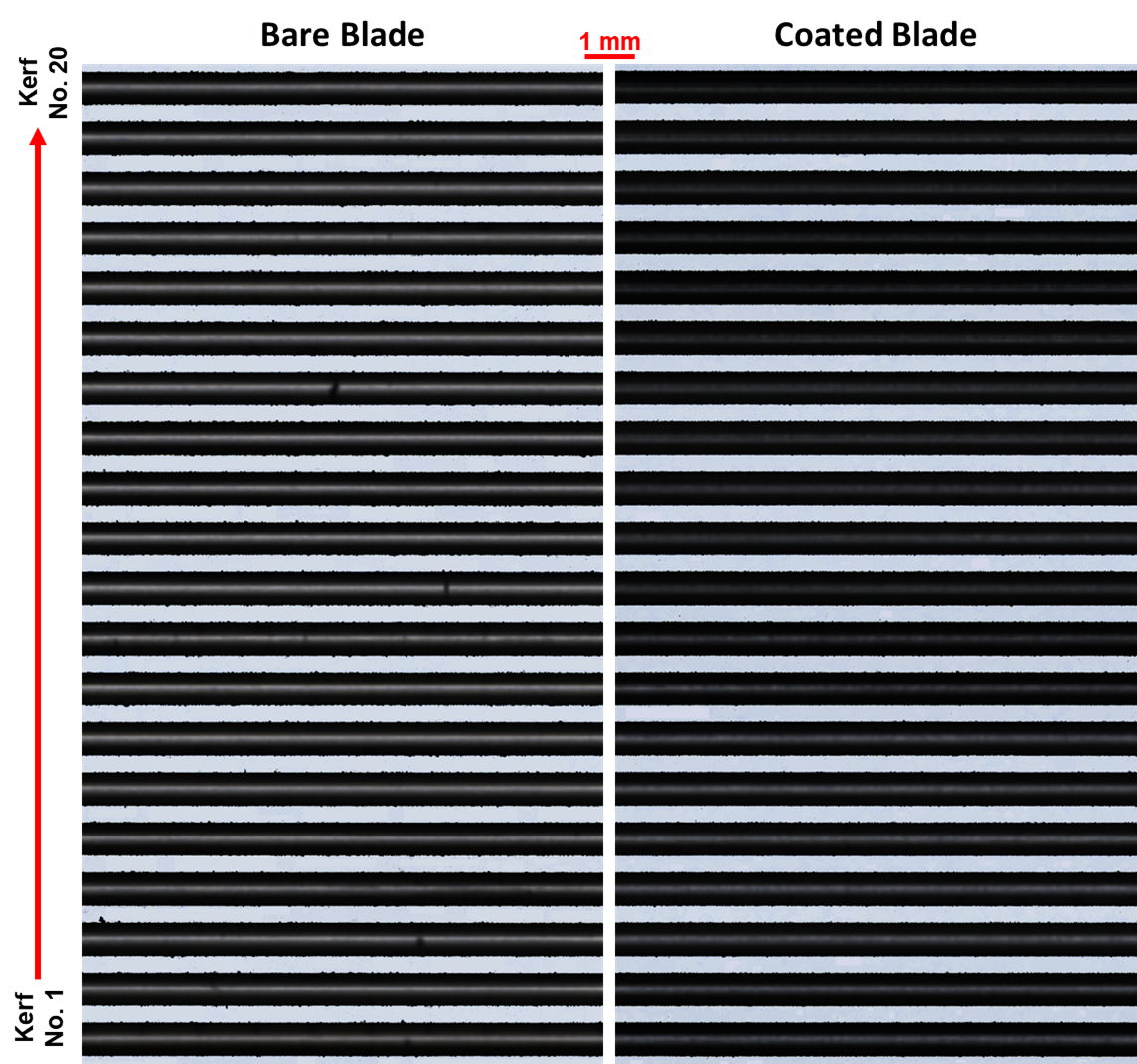
**

**Supplementary Figure S1.** Laser confocal micrographs of 20-kerf Si workpiece after dicing with bare and coated blades.


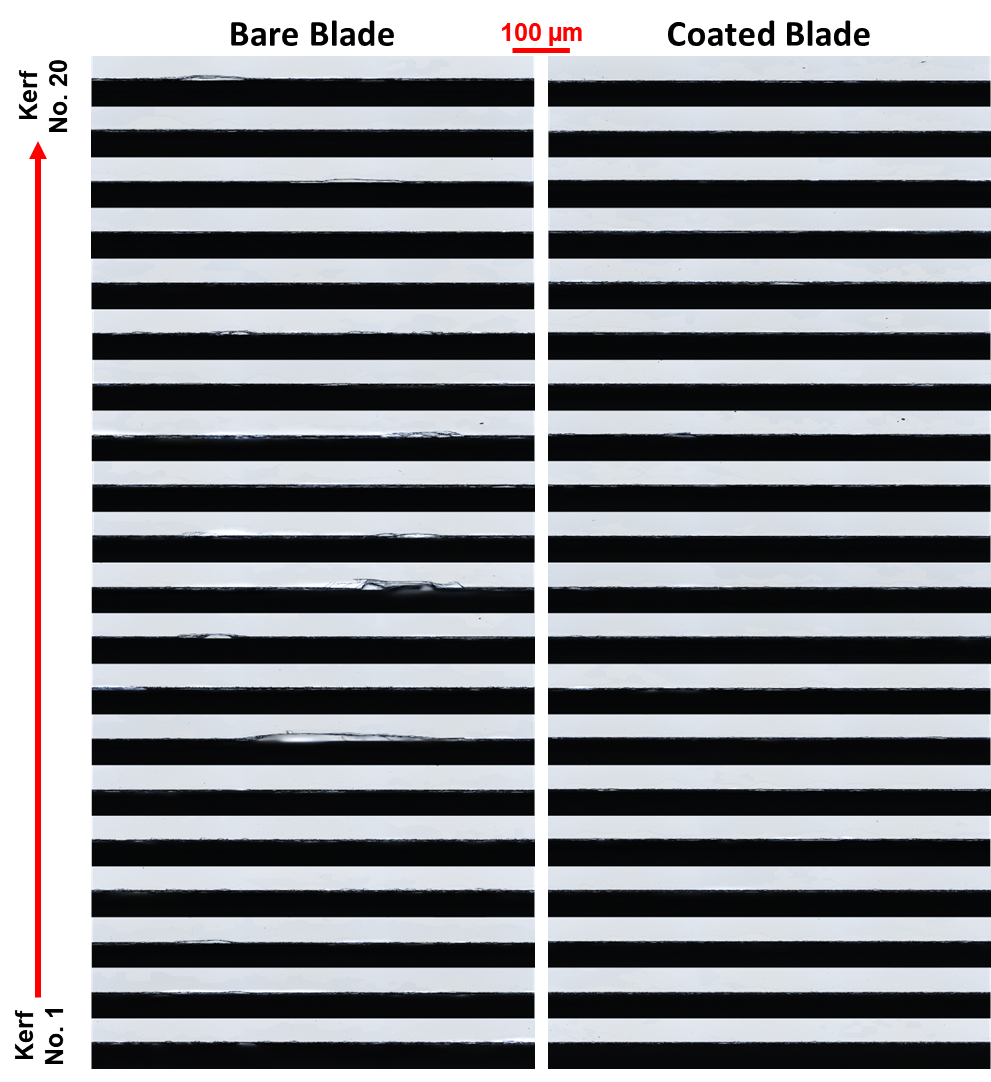


**Supplementary Figure S2(a)**. Laser confocal micrographs of 20-kerf SiC workpiece in upper edge regions after dicing with bare and coated blades. The DISCO blade was used in this case.


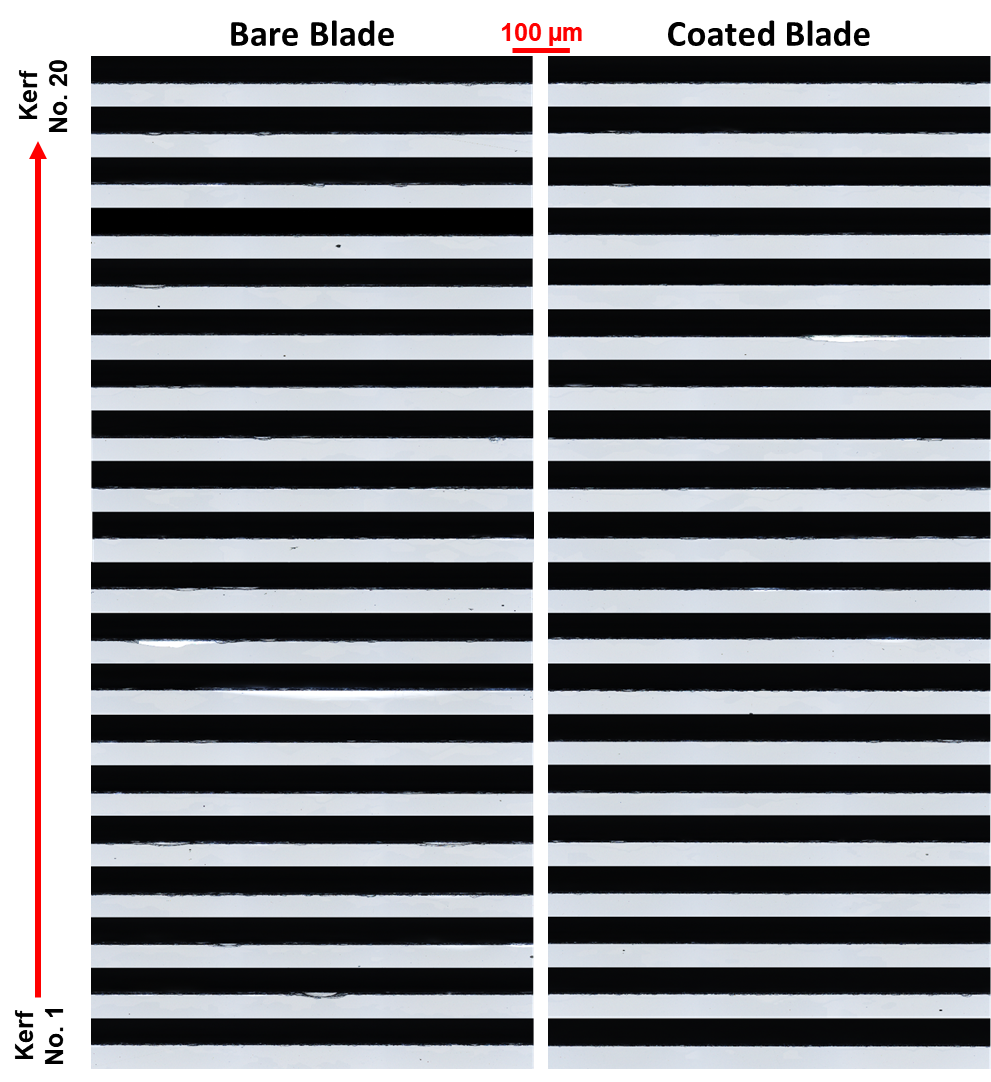


**Supplementary Figure S2(b)**. Laser confocal micrographs of 20-kerf SiC workpiece in lower edge regions after dicing with bare and coated blades. The DISCO blade was used in this case.


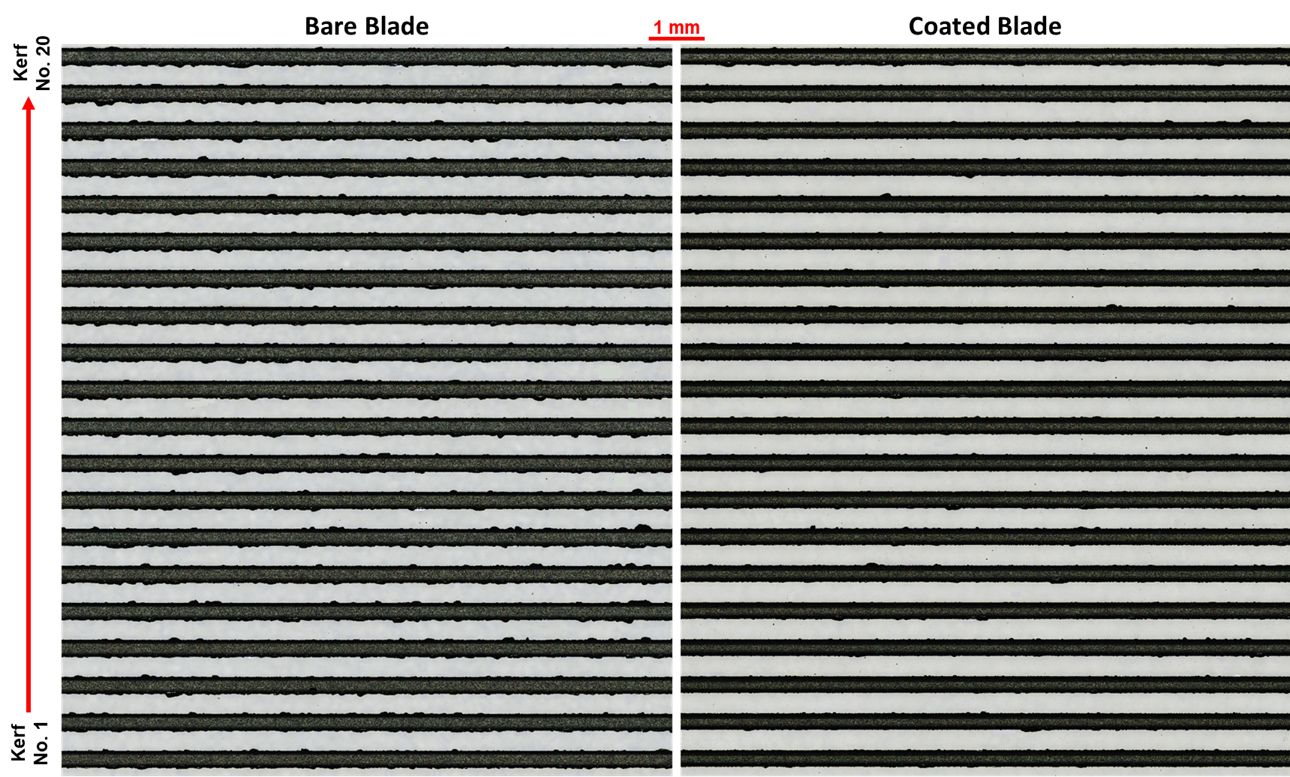


**Supplementary Figure S3**. Laser confocal micrographs of 20-kerf sapphire workpiece after dicing with bare and coated blades.

**
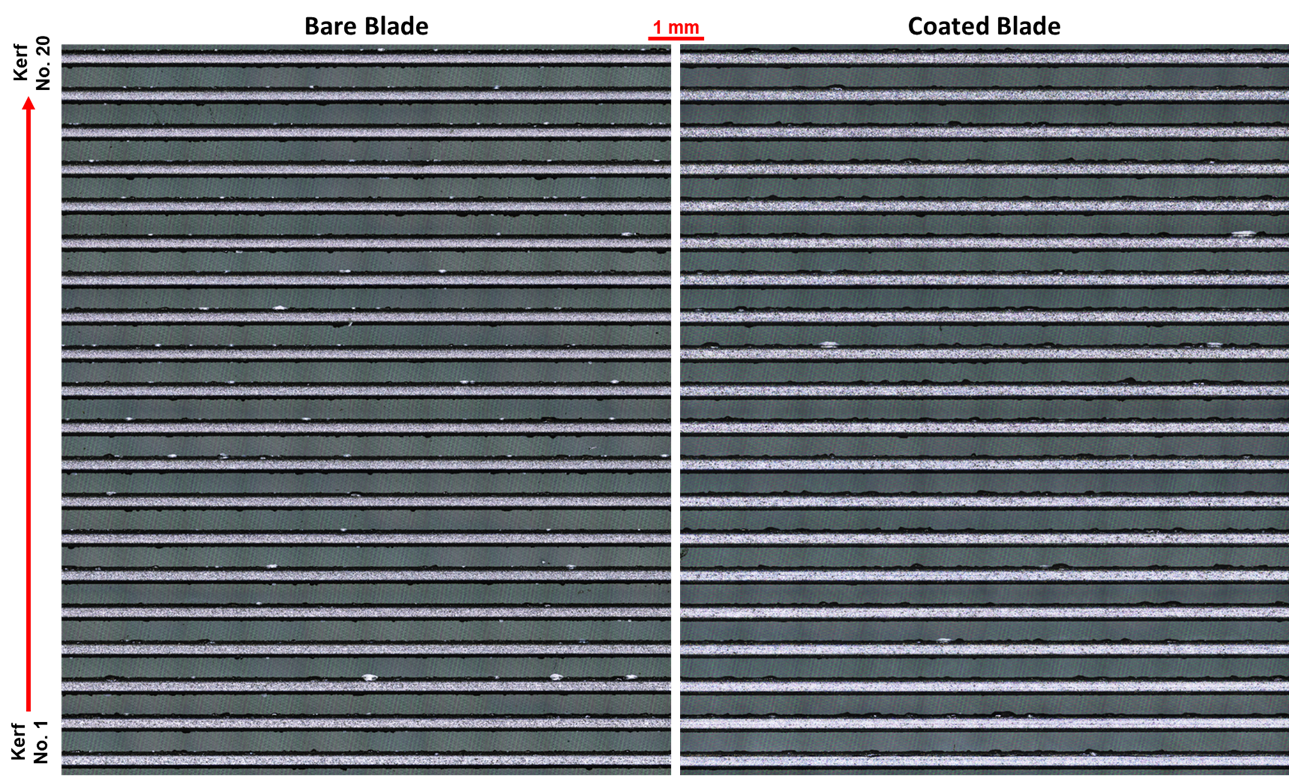
**

**Supplementary Figure S4**. Laser confocal micrographs of 20-kerf PSS workpiece after dicing with bare and coated blades.
